# The small things can matter

**DOI:** 10.1371/journal.pbio.3000009

**Published:** 2018-08-24

**Authors:** Norman R. Pace

**Affiliations:** Molecular, Cellular, and Developmental Biology, University of Colorado, Boulder, Colorado, United States of America

## Abstract

In the context of biology as a whole and of our own personal lives, seemingly small things can prove surprisingly influential. Here, I consider the powerful impact of small organisms—the inhabitants of the microbial world—and the small events that shaped my own development as a scientist. I reflect on the early days of the fields of molecular biology and microbial ecology and my own role in the origin story of what we now call “metagenomics”.

## Abstract 2

This Research Matters article remembers the early days of what became known as "metagenomics," as experienced by the author during his development as a scientist.

Classical biologists, already confronted with bewildering diversity among large organisms, tended to ignore those they couldn’t see. Microbes were considered significant mostly in the context of disease and rot, despite many hints of their deeper importance to humans. We knew about the value of antibiotics and other fermentation products and that microbial symbiosis with some plants results in nitrogen fixation. But the identification of specific diseases caused by microbes molded a public perception of a malevolent, at least yucky, microbial world. Only in recent decades has the microbial world been recognized as a driver of global biology, deeply intertwined with the health and functions not only of ourselves but of the biosphere at large. How, in retrospect, could such a conspicuous influence on the biosphere have been largely ignored?

There were many reasons for the general biologist to ignore microbes. First of all, observing them requires special equipment, such as microscopes. Then, there’s the question of how to distinguish and classify the myriad microscopic forms seen in a pinch of any environmental sample—a question that has vexed microbiologists since scientists discovered microbes in the 17th century and developed culture-based methods to describe specific organisms. However, as long recognized by microbiologists, only a small fraction, perhaps one in 1,000 of microscopically observable organisms in environmental samples, is cultured using standard techniques. Yet even among the culturable fraction, few characteristics could be used to identify different microbes specifically. Through the 1970s, formal taxonomy of most microbes was crude and inaccurate, generally relying on gross morphology, anecdotal nutritional properties, and much speculation. Microbes typically defied an evolutionary taxonomy in the traditional sense because they lacked relatable properties.

## Woese to the rescue

This state of affairs changed in the 1970s with Carl Woese’s focus on comparisons of ribosomal RNA sequences and the resulting discovery of the fundamental phylogenetic organization of all biology [1]. There are three rRNAs: “5S” rRNA (120 nucleotides [NTs]), “16S” rRNA (1,500–2,000 NTs) and “23S” rRNA (3,000–5,000 NTs). Although sequencing large RNAs was impossible when Woese undertook these studies, he developed clever ways to determine sequences of small fragments of 16S rRNA, many of which were useful for interspecies comparisons. With this rudimentary sequence information, Woese was able to infer the basic structure of an evolutionary tree of life consisting of bacteria, archaea, and eukaryotes; he could begin to make sequence-based maps of evolutionary relationships—“phylogenetic trees”.

An rRNA sequence-based phylogenetic tree is a specific coordinate system for positioning the rRNA sequence of any organism in the tree. The scale is sequence difference; the positional coordinate for any organism is relative to known rRNA sequences. For the first time, an analytical approach could be brought to bear on the phylogenetic identification of otherwise unknown, even uncultured, microorganisms. Sequence-based phylogenetic trees also begin to answer some long-standing questions: How should “biological diversity” be described? How “different” are different organisms? What were the paths of evolution, and where in the tree of life was the origin of cellular evolution?

## Early inspiration

I came into questions of this nature through the small happenstances that occur to everyone while growing up. My own background was in a small farming community in eastern Indiana. I had an early interest in microbial things, and my parents encouraged my scientific bent with a microscope kit. It wasn’t a very good microscope, but even at low magnifications, I could see lots of really strange things that weren’t described in the biology texts. I couldn’t do anything with that world. I did some simple experiments growing molds on various media, but overall, nothing came from it except interest in this peculiar world. I also during this period developed an interest in chemistry and had a fairly elaborate laboratory.

One important thing that molded my career, seemingly small to me at the time, happened in October 1957. I was a paperboy then and recall one dawn cutting the wire on my bundle of papers and seeing the headline “Reds Launch Satellite!” As a bit of a space and sci-fi buff, as much as a kid in the 1950s could be, I found this interesting, even exciting, but not personally relevant. Not true, as it turned out.

Congress took note of the Soviet advance and decided that United States kids needed to be drawn into science. One of the implementations was funding of a series of “High School Science Institutes” at universities around the country. At Indiana University, 64 students between their junior and senior years in high school were selected from around the state and brought to Indiana University for two weeks of lectures and lab exercises. I was chosen for reasons still unclear to me. At the end of the initial period, some of us were posted to university labs for an additional two months of work. I was assigned, I guess arbitrarily, to the lab of Dean Fraser, an early phage and molecular biologist (before the term “molecular biology” was invented). I worked with a graduate student and a small paper resulted. More importantly to me was the exposure to the university world and academic science. I was hooked. This led me to undergraduate and graduate degrees in microbiology, microbiology because that’s where you went for molecular biology then. I learned a lot about physiology and rudimentary molecular biology. Microbial ecology was largely anecdotal and had not yet developed as a field. At the same time, I developed a lifelong involvement in cave exploration and mapping. I realized that there are new things to be discovered, often just underfoot.

My focus in graduate school was in vitro phage RNA replication, and my early career was built around the molecular biology of RNA processing and RNA chemistry. A small thing at the time, critical in the longer term, was that Woese served on my thesis committee at the University of Illinois and we became friends. This was prior to his celebrated work using rRNA sequences to infer phylogenetic relationships, which I was able to watch develop and be involved with in a small way.

## From molecules to microbes

My path into microbial ecology was through RNA processing, mechanisms through which large precursor RNAs are cleaved or otherwise modified to form mature, functional RNAs. My lab had developed an in vitro system for maturation of 5S rRNA to the point that further understanding of enzymological details would require solving the crystal structure. This was before the availability of in vitro transcription systems for making the milligram quantities of 5S RNA needed for crystallization, so I wondered if some naturally occurring microbial biomass might be an abundant and convenient source of RNAs.

While studying potential sources, I encountered in Brock’s *Thermophilic Microorganisms and Life at High Temperatures* the near-boiling Yellowstone hot spring “Octopus Spring,” containing large quantities of “pink filaments,” aggregates of hyperthermophilic microbes of unknown kind. Despite many efforts, Brock and colleagues were unable to culture and thereby classify the pink filaments using the usual culture-dependent methodologies. Identification of an organism of study is expected protocol. However, I reasoned that we could sidestep any expectation for classical identification by using a molecular approach: rRNA sequence comparisons to establish a “phylogenetic identification” in the context of the developing Woese reference framework. This sort of identification would be far more incisive than the classical microbial taxonomy. An rRNA sequence provides a coordinate in phylogenetic space relative to comparable sequences from known organisms. With this perspective, culture of a microbe was no longer a prerequisite for identification. My colleagues David Stahl, Gary Olsen, David Lane, and I all immediately recognized the broad potential of this notion. It meant that the makeup of the natural microbial world could be described independently of that crippling requirement for culture to identify individual microbes.

This was 1981. PCR was not yet invented. Machine sequencing was not yet invented. You had to make your own restriction enzymes. You had to synthesize your own oligonucleotides, as they became useful. DNA-cloning and facile-cloning vectors were just on the table. There were no sequence databases, indeed few gene sequences of any kind had been determined; computers were rudimentary, and the World Wide Web didn’t exist. Nonetheless, with bootlegged support from my molecular biology grants, we undertook to develop the use of molecular technology based on rRNA sequences to characterize the makeup of natural microbial communities phylogenetically.

## New tools open the door to environmental microbial diversity

We demonstrated the utility of the approach using 5S rRNA and RNAs from sulfide-oxidizing symbionts associated with the deep-sea hydrothermal vent invertebrate *Riftia pachyptila* [2] and also from Octopus Spring [3]. That RNA, although only approximately 120 NTs, was sufficiently small that it could be isolated by high-resolution gel electrophoresis from mixed-organism communities and sequenced. Based on comparison with known sequences, the *Riftia* symbiont proved a close relative of cultured organisms, *Thiomicrospira* spp. Nothing particularly new. The main Octopus Spring sequences and their contributing organisms, however, were not specifically related to any known organisms. We knew, then, that there was much to discover and that the natural microbial world was accessible to us through culture-independent molecular phylogenetic analyses.

Although we used 5S rRNA sequences for some further studies, the more useful phylogenetic marker was the larger 16S rRNA to interface with the growing collection of Woese’s partial sequences. The problem was that there were no 16S rRNA sequences known beyond that of *Escherichia coli*, recently determined by Brosius and Noller. In order to accumulate sequences of various organisms expeditiously, we developed a series of short sequences based on the Woese catalogs and the *E*. *coli* sequence and used those as “universal” primers for reverse transcription sequencing of conveniently isolated 16S rRNA [4]. When PCR became available, these primers became the basis for specific amplification of rRNA genes for sequence analysis and are still widely used.

So we could now identify environmental organisms phylogenetically, but how to complete the circle and identify morphotypes in the environment with the rRNA sequences? This we solved by synthesizing fluorescently labeled oligonucleotides complementary to test rRNA sequences. Because there are many thousands of ribosomes in all cells, detection of fluorescent probe binding to single cells could be readily detected by UV microscopy and specific organisms related to specific sequences. We termed these tools “phylogenetic stains” to acknowledge the long importance of specific microscopic stains to the study of microbiology [5].

The final step in the program we had planned was the application of “shotgun” cloning to recover environmental genes from complex communities [6]. We chose to study marine “picoplankton,” microbes from the open ocean that seemed microscopically ubiquitous but about which essentially nothing was known. At that stage, cloning genes required natural DNA—a lot of it. To gather the requisite DNA, Tom Schmidt and Ed DeLong filtered approximately 8,000 L of low-nutrient Pacific Ocean water off Hawaii. DNA from the resulting few hundred mg of picoplankton biomass was used to construct 20-kb inserts into phage lambda DNA; rRNA gene-containing clones were identified for sequencing by hybridization with radioactive “universal” oligonucleotide probes [5]. Of course, we realized that any other environmental gene could be obtained the same way, from the same recombinant library. The way into the natural microbial world was open.

Woese’s rRNA-based phylogenetic taxonomy and the application of culture-independent molecular methods to exploit that perspective drew many investigators worldwide. Knowledge of microbial diversity exploded. For instance, by the mid-1980s, approximately 12 bacterial phyla were known; by 2005, approximately 100 phyla had been seen in molecular surveys, and currently, far more have been detected. Even a phylogenetic definition of what distinguishes bacterial phyla is now elusive. Microbial ecology has matured enormously, and the precepts have even made deep inroads into understandings and contributions to human health. And this is only a small beginning, as we learn more about how to live with and make use of that still poorly understood microbial world. The Golden Age of microbiology is still ahead of us.

**Image 1:**
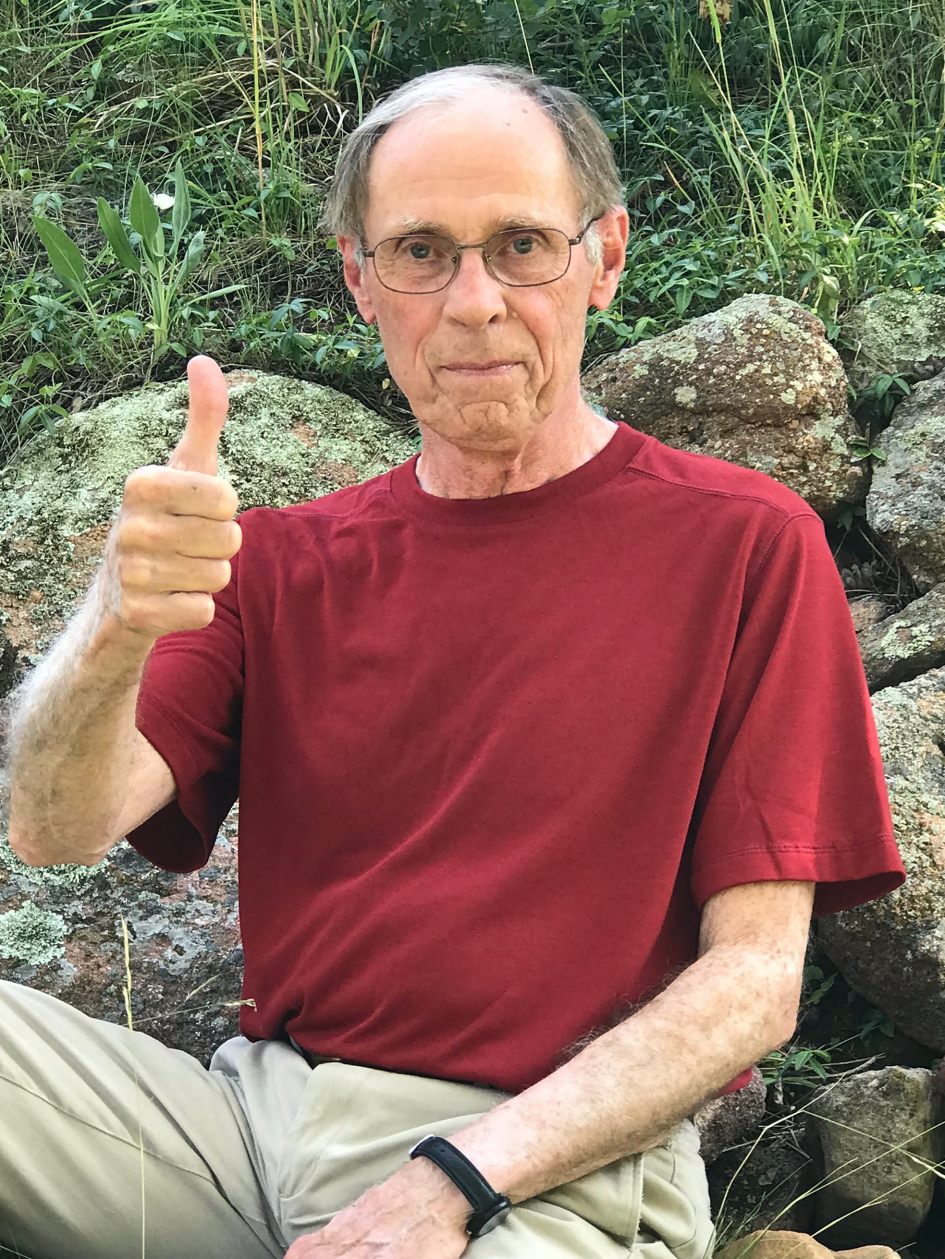
Norman R. Pace.

## Abbrevation

NT: nucleotide
